# Ionic Environment Affects Biomolecular Interactions of Amyloid-β: SPR Biosensor Study

**DOI:** 10.3390/ijms21249727

**Published:** 2020-12-20

**Authors:** Erika Hemmerová, Tomáš Špringer, Zdeňka Krištofiková, Jiří Homola

**Affiliations:** 1 Institute of Photonics and Electronics of the Czech Academy of Sciences, Chaberská 1014/57, 182 51 Prague, Czech Republic; hemmerova@ufe.cz (E.H.); springer@ufe.cz (T.Š.); 2 National Institute of Mental Health, Topolová 748, 250 67 Klecany, Czech Republic; zdenakristofikova@gmail.com

**Keywords:** biomolecular interactions, mitochondrial matrix, surface plasmon resonance (SPR), amyloid beta (Aβ), cyclophilin D (cypD), 17β-hydroxysteroid dehydrogenase 10 (17β-HSD10), ionic environment

## Abstract

In early stages of Alzheimer’s disease (AD), amyloid beta (Aβ) accumulates in the mitochondrial matrix and interacts with mitochondrial proteins, such as cyclophilin D (cypD) and 17β-hydroxysteroid dehydrogenase 10 (17β-HSD10). Multiple processes associated with AD such as increased production or oligomerization of Aβ affect these interactions and disbalance the equilibrium between the biomolecules, which contributes to mitochondrial dysfunction. Here, we investigate the effect of the ionic environment on the interactions of Aβ (Aβ_1–40_, Aβ_1–42_) with cypD and 17β-HSD10 using a surface plasmon resonance (SPR) biosensor. We show that changes in concentrations of K^+^ and Mg^2+^ significantly affect the interactions and may increase the binding efficiency between the biomolecules by up to 35% and 65% for the interactions with Aβ_1–40_ and Aβ_1–42_, respectively, in comparison with the physiological state. We also demonstrate that while the binding of Aβ_1–40_ to cypD and 17β-HSD10 takes place preferentially around the physiological concentrations of ions, decreased concentrations of K^+^ and increased concentrations of Mg^2+^ promote the interaction of both mitochondrial proteins with Aβ_1–42_. These results suggest that the ionic environment represents an important factor that should be considered in the investigation of biomolecular interactions taking place in the mitochondrial matrix under physiological as well as AD-associated conditions.

## 1. Introduction

Alzheimer’s disease (AD) is currently the most common neurodegenerative disease of the elderly. It is characterized by extensive neuronal failure in which, according to the current understanding of AD, peptide amyloid beta (Aβ) plays a central role. Aβ is most commonly expressed as Aβ_1–40_ and Aβ_1–42_ fragments that are comprised of 40 and 42 amino acids, respectively [1]. During AD, the production of Aβ is increased and skewed towards Aβ_1–42_ [2]. Both fragments of Aβ are known to form oligomers (Aβ_1–42_ more readily than Aβ_1–40_ [3,4]) which have been shown to cause more substantial neuronal damage than monomers [5]. However, the exact molecular mechanisms of Aβ-induced pathological processes during AD remain poorly understood. Multiple hypotheses have been proposed [6], such as the mitochondrial cascade hypothesis [7], amyloid toxic oligomer hypothesis [8], calcium hypothesis [9] and lipid-chaperon hypothesis [10]. In this work, we follow the mitochondrial cascade hypothesis and investigate the Aβ-triggered processes taking place in mitochondria during AD.

In early stages of AD, Aβ accumulates in the mitochondrial matrix, where it interacts with multiple mitochondrial proteins, such as cyclophilin D (cypD) [11,12] and 17β-hydroxysteroid dehydrogenase type 10 (17β-HSD10) [13,14]. These interactions have been suggested to form a biomolecular link between Aβ and deteriorated mitochondrial functions [11,14], such as decreased energy metabolism [15,16], increased production of reactive oxygen species (ROS) [17] or formation of mitochondrial permeability transition pores (mPTPs) [12,18]. 

Interaction properties of Aβ have been the subject of multiple studies; in in vitro studies, synthetic Aβ oligomers generated in vitro have commonly been used [11,13,19,20]. Although there are differences in the biological characteristics of natural (obtained from biological samples) and synthetic oligomers, the differences are mainly in the efficiency of their action rather than in their function in biological processes [21,22]. In our recent work, we studied interactions of Aβ with cypD and 17β-HSD10, and with the use of a multi-interaction model, we demonstrated that the processes associated with early stages of AD such as the oligomerization of Aβ and increased/favored production of Aβ_1–42_ affect the equilibrium between these biomolecules in the mitochondrial matrix [23]. We also showed that 17β-HSD10 specifically binds cypD and thus regulates levels of free cypD in the mitochondrial matrix [24,25]. Our study also revealed that Aβ disrupts this regulation [24] which may result in the translocation of free cypD to the inner mitochondrial membrane and induce the formation of mPTPs [15]. In addition, our study showed that the ability of 17β-HSD10 to regulate cypD is significantly affected by the ionic environment in which the interaction takes place [24].

The ionic environment inside the mitochondrial matrix is established via multiple specialized channels through which ions are transferred between the cytosol and mitochondrial matrix. Physiological and pathological factors and processes (e.g., the temporal metabolic situation of the cell or the perturbations in the cytosolic environment) regulate this transfer and thus the concentrations of ions inside the mitochondrial matrix [26,27,28,29,30]. The most abundant ion present in the mitochondrial matrix is K^+^ which regulates/is regulated by the mitochondrial volume and the level of produced ROS [31,32]. It is assumed that K^+^ is present at a concentration of 140 mM both in the matrix and cytosol [31,33]. However, it has also been demonstrated that a decrease in the membrane potential (e.g., during the synthesis of ATP) may induce a decrease in K^+^ concentrations [34] and there are works that have reported considerably lower K^+^ concentrations (down to 15 mM) [32,35]. Mg^2+^ is the most abundant bivalent ion in the mitochondrial matrix and is typically present at concentrations of about 15–18 mM [36]. The majority of Mg^2+^ is bound by ATP and other Mg^2+^-binding biomolecules for which it serves as a cofactor [29] and only approximately 0.5–2.5 mM of the total Mg^2+^ is free [28,37]. In response to stimuli, such as the presence of particular hormones [26], increased energy demands of the cell [37] or perturbations in ionic strength inside the mitochondrial matrix [26], Mg^2+^ may dissociate from the complexes with biomolecules or leak from the matrix to the cytosol and thus increase or decrease the concentration of free Mg^2+^ in the mitochondrial matrix, respectively [38]. Moreover, it seems that the actions of different ions are interconnected and changes in the concentrations of one ion influence the distribution of the others [34]. In addition, the net ionic strength is also likely to fluctuate. Despite the number of studies on the ionic environment in the mitochondrial matrix, the knowledge of concentration ranges of ions and their relationship to particular biomolecular processes remains limited.

In this work, we investigate the effect of the ionic environment on the interactions of Aβ (Aβ_1–40_, Aβ_1–42_) with cypD and 17β-HSD10. The oligomeric forms of Aβ_1–40_ and Aβ_1–42_ pertinent to AD, i.e., monomeric Aβ_1–40_ and oligomeric Aβ_1–42_, are considered. In particular, we study the biomolecular interactions in the presence of K^+^ and Mg^2+^ using the surface plasmon resonance (SPR) biosensor method and show how changes in the concentrations of these ions affect the biomolecular interactions.

## 2. Results

### 2.1. Interactions of Aβ_1–40_ and Aβ_1–42_ with cypD at Different Concentrations of K^+^ and Mg^2+^

**Figure 1:**
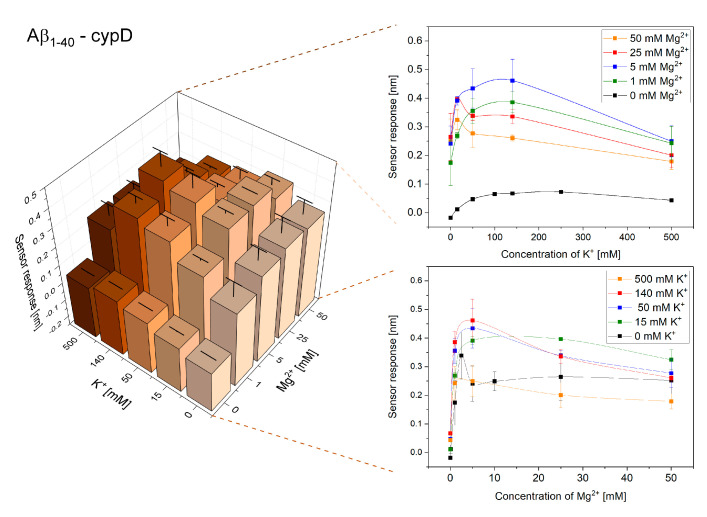
Reference-compensated sensor response to the binding of monomeric Aβ_1–40_ to the immobilized cypD as a function of concentration of K^+^ and Mg^2+^.

**Figure 2:**
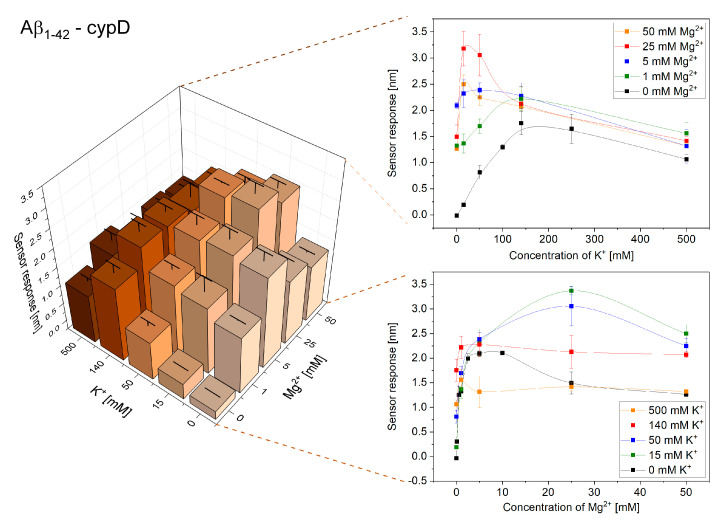
Reference-compensated sensor response to the binding of oligomeric Aβ_1–42_ to the immobilized cypD as a function of concentration of K^+^ and Mg^2+^.

Initially, we investigated the effect of varying concentrations of K^+^ and Mg^2+^ on the interactions of cypD and Aβ. Figure 1 and Figure 2 show the obtained reference-compensated sensor responses corresponding to the binding of monomeric Aβ_1–40_ and oligomeric Aβ_1–42_ to the immobilized cypD in the presence of different concentrations of K^+^ and Mg^2+^, respectively, and demonstrate the significant effect of these ions on the interactions. 

The binding efficiency of the interactions follows a complex trend. The binding efficiency is lowest in the absence of K^+^ and Mg^2+^, increases with increasing concentrations of ions until it reaches the maximum and then decreases. The maximum binding efficiency of the interactions between cypD and Aβ occurred at different ionic concentrations for Aβ_1–40_ and Aβ_1–42_. While the highest binding efficiency of the interaction between cypD and Aβ_1–40_ was observed at 140 mM K^+^ and 5 mM Mg^2+^, the highest binding efficiency of the interaction between Aβ_1–42_ and cypD occurred at 15 mM K^+^ and 25 mM Mg^2+^.

The different effects of ions on the binding efficiency between Aβ and cypD observed for Aβ_1–40_ and Aβ_1–42_ may originate from two main factors: (1) the different oligomerization state of the particular fragments of Aβ (Aβ_1–40_ in the monomeric form, Aβ_1–42_ in the oligomeric form), and (2) the structural differences between the two fragments (two additional hydrophobic amino acids in the structure of Aβ_1–42_). In order to evaluate the contribution of these factors, we investigate the effect of the oligomerization state of Aβ on the interactions (Section 2.2).

### 2.2. The Effect of the Oligomerization State of Aβ 

In order to evaluate whether the different effects of ions on the binding between Aβ and cypD observed for Aβ_1–40_ and Aβ_1–42_ may originate from the different oligomerization states of the particular fragments of Aβ, we analyzed the binding of Aβ_1–40_ and Aβ_1–42_ in both monomeric and oligomeric forms (see Section 4.1.3 in Materials and Methods) to the immobilized cypD. The binding was observed in the presence of 5 mM Mg^2+^ and varying concentrations of K^+^, as under these conditions, the differences in trends obtained for the different fragments of Aβ were the most evident.

**Figure 3:**
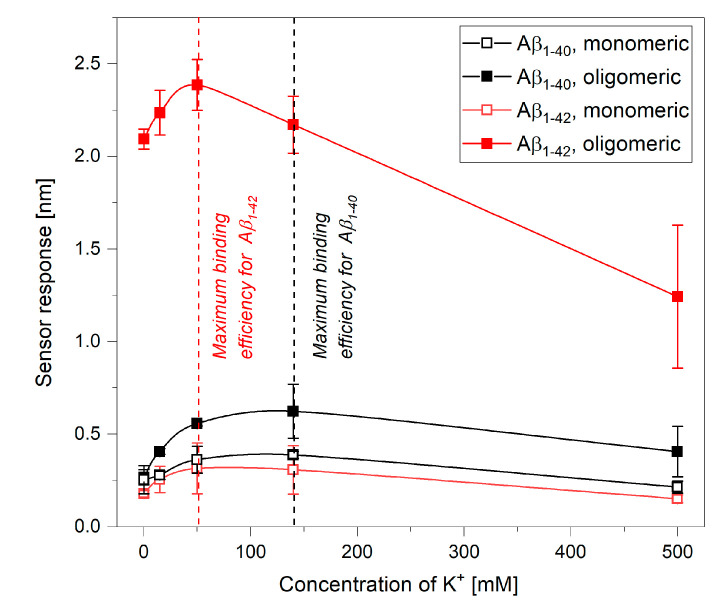
Reference-compensated sensor response to the binding of Aβ in different oligomerization forms to the immobilized cypD as a function of concentration of K^+^ in the presence of 5 mM Mg^2+^.

Figure 3 shows that oligomeric forms of Aβ exhibit higher sensor responses in comparison to the monomeric forms, which agrees well with the results of our previous study [14]. However, the maximum binding efficiency occurs for the same ionic environment for both oligomerization forms for both fragments of Aβ. It occurs in the presence of 140 mM K^+^ for the interaction between monomeric as well as oligomeric Aβ_1–40_ and cypD and in the presence of 50 mM K^+^ for the interaction between monomeric as well as oligomeric Aβ_1–42_ and cypD. Thus, we conclude that the observed trends are not affected by the different oligomerization states of the fragments but rather originate from the structural differences between Aβ_1–40_ and Aβ_1–42_.

### 2.3. Interactions of Aβ_1–40_ and Aβ_1–42_ with 17β-HSD10 at Different Concentrations of K^+^ and Mg^2+^

**Figure 4:**
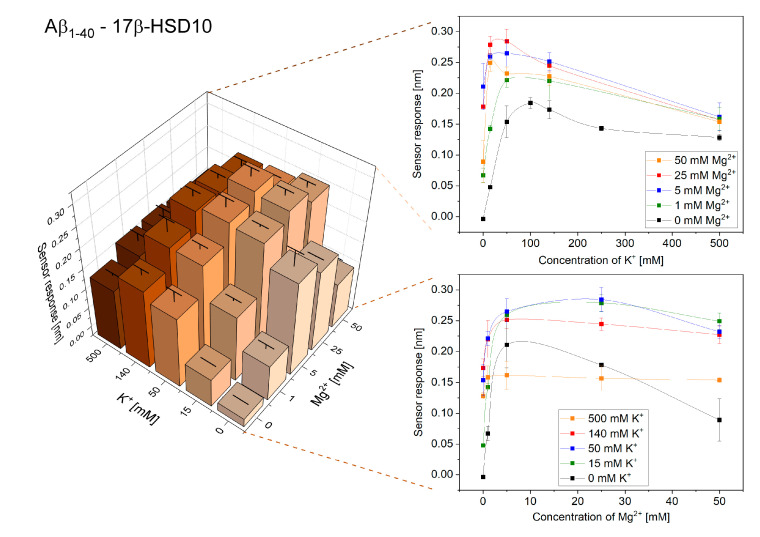
Reference-compensated sensor response to the binding of monomeric Aβ_1–40_ to the immobilized 17β-HSD10 as a function of concentration of K^+^ and Mg^2+^.

**Figure 5:**
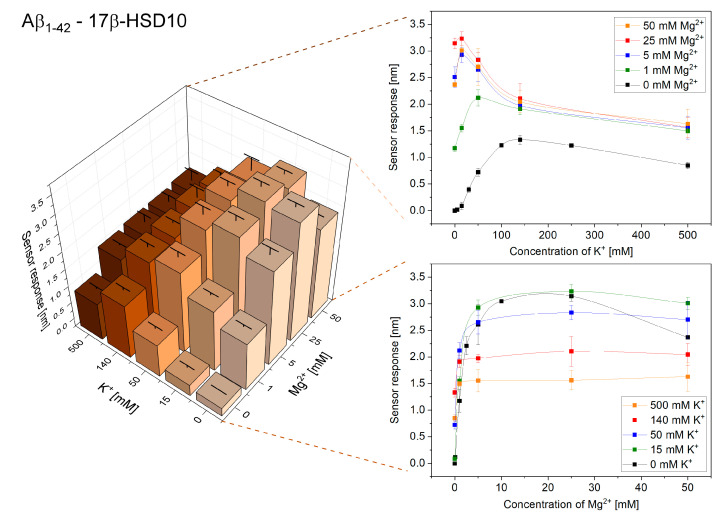
Reference-compensated sensor response to the binding of oligomeric Aβ_1–42_ to the immobilized 17β-HSD10 as a function of concentration of K^+^ and Mg^2+^.

In this part of the study, we investigated the effect of the ionic environment on the interactions of 17β-HSD10 and Aβ. Figure 4 and Figure 5 show the obtained reference-compensated sensor responses to the binding of monomeric Aβ_1–40_ and oligomeric Aβ_1–42_ to the immobilized 17β-HSD10 in the presence of varying concentrations of K^+^ and Mg^2+^, respectively. 

We observed a peak in the efficiency of the binding between 17β-HSD10 and Aβ for ionic compositions that were different for Aβ_1–40_ and Aβ_1–42_ (similarly as for the interaction between cypD and Aβ). The maximum binding efficiency for the interaction of 17β-HSD10 with Aβ_1–40_ and Aβ_1–42_ was observed for 50 mM K^+^ and 25 mM Mg^2+^ and for 15 mM K^+^ and 25 mM Mg^2+^, respectively.

## 3. Discussion

Our results demonstrate that perturbations of the concentrations of K^+^ and Mg^2+^ may considerably affect the interactions between biomolecules in the mitochondrial matrix (Figure 1, Figure 2, Figure 3, Figure 4 and Figure 5). For all the interactions investigated herein, we observed a peak in the maximum binding efficiency occurring at a particular ionic composition. The peak was sharper for interactions of Aβ_1–42_ with both cypD and 17β-HSD10 when compared to the interactions of Aβ_1–40_, suggesting that the interactions of Aβ_1–42_ are more sensitive to changes in concentrations of ions. Moreover, the position of the peak differed for different interactions. It occurred at 15 mM K^+^ and 25 mM Mg^2+^ for interactions of Aβ_1–42_ and at 50–140 mM K^+^ and 5–25 mM Mg^2+^ for interactions of Aβ_1–40_ with both cypD and 17β-HSD10. Assuming the physiological concentrations of ions in the mitochondrial matrix to be 140 mM K^+^ and 1 mM Mg^2+^, we conclude that the binding efficiency of the interactions of Aβ with cypD and 17β-HSD10 may increase by 20–35% for Aβ_1–40_ and by 40–65% for Aβ_1–42_ in comparison with the physiological conditions.

In our previous study, we showed that processes related to AD such as increased production of Aβ, unbalanced production of Aβ fragments favoring Aβ_1–42_ or oligomerization of Aβ_1–42_ significantly affect the interactions between Aβ and mitochondrial proteins and enhance the binding of Aβ_1–42_ to both cypD and 17β-HSD10 [23]. In this work, we demonstrate that the mitochondrial ionic environment also plays an important role in biomolecular processes taking place in the mitochondrial matrix, and we show that the conditions causing decreased concentrations of K^+^ and increased concentrations of Mg^2+^ may further enhance the binding of Aβ_1–42_ to both cypD and 17β-HSD10.

Despite the large number of studies on the ionic transport in mitochondria, it remains difficult to relate a specific ionic composition to a particular process taking place in the mitochondrial matrix. Due to the oxidative inactivation of ATP-related enzymes, decreased production of ATP has been observed in affected areas of the AD brain [39]. Bredshaw et al. associated the decreased levels of ATP with the increased concentration of free Mg^2+^ inside the mitochondrial matrix [26]. The excess of free Mg^2+^ may inhibit the function of mitoK_ATP_, which may decrease the influx of K^+^ in mitochondria [40]. Therefore, we hypothesize that, under the circumstances of progressing AD, the deteriorated mitochondrial functions alter the ionic environment in mitochondria which promotes the interactions of Aβ_1–42_ with cypD and 17β-HSD10. These interactions lead to the inhibition of the enzymatic function of 17β-HSD10 [14] and contribute to an increased production of ROS [41] and thus further aggravate the pathology of AD.

## 4. Materials and Methods 

### 4.1. Materials

#### 4.1.1. Reagents

NaCl, NaOH, KCl, MgCl_2_, hexafluoroisopropanol (HFIP), NH_4_OH, bovine serum albumin (BSA) and all buffers were purchased from Sigma-Aldrich, Czech Republic. Thiols: 11-mercapto-hexa(ethyleneglycol)undecyloxy acetic acid (HS-C_11_-(EG)_6_-OCH_2_-COOH) and 11-mercapto-tetra(ethyleneglycol)undecanol (HS-C_11_-(EG)_4_-OH), were purchased from Prochimia, Poland. *N*-hydroxysuccinimide (NHS), 1-ethyl-3-(3-dimethylaminopropyl)-carbodiimide hydrochloride (EDC) and ethanolamine hydrochloride (EA) were purchased from GE Healthcare, Sweden. 17β-HSD10 (human, recombinant), cypD (human, recombinant) and an antibody against cypD (Ab(cypD)) were purchased from Fitzgerald, USA. An antibody against 17β-HSD10 (Ab(17β-HSD10)) was purchased from Biolegend, USA. Aβ_1–40_ and Aβ_1–42_ (human, synthetic) were obtained from AnaSpec, USA, dissolved in 1% NH_4_OH and diluted by PBS to obtain the stock concentration of 100 μM.

#### 4.1.2. Buffers

The buffers used for the functionalization of SPR chips and the immobilization of cypD and 17β-HSD10 to the surface included the following: sodium acetate (SA10; 10 mM, pH 5.0), MES (10 mM, pH 5.0), phosphate-buffered saline (PBS; 10 mM phosphate, 2.7 mM KCl, 138 mM NaCl, pH 7.4), high-ionic strength PBS (PBS_Na_; 10 mM phosphate, 2.7 mM KCl, 750 mM NaCl, pH 7.4). All the buffers were prepared using deionized Milli-Q water (Merck, Czech Republic).

The running buffer (RB) with varying concentrations of K^+^ and Mg^2+^ was used for the binding of Aβ to the surface to simulate different ionic environments of the interactions of Aβ with cypD and 17β-HSD10. All the used RBs were prepared as 10 mM HEPES in Milli-Q with addition of BSA (200 μg/mL). The pH was adjusted by NaOH to 7.4 and NaCl was used to set the concentration of Na^+^ to 5 mM. Concentrations of K^+^ and Mg^2+^ in RBs were adjusted by the addition of aqueous solutions of KCl and MgCl_2_ at particular concentrations that ensured the same dilution (9:1) of RBs for all the concentrations of ions.

#### 4.1.3. Preparation of Aβ Samples

In this work, Aβ_1–40_ and Aβ_1–42_ fragments with different oligomerization states were prepared using the procedures described in our previous study [23]. The oligomeric forms of Aβ_1–40_ and Aβ_1–42_ were prepared using Preparation A, whereas monomeric Aβ_1–40_ and monomeric Aβ_1–42_ were prepared using Preparation C and Preparation B, respectively. Briefly, in Preparation A, Aβ from the stock solution was diluted by RB to obtain the final concentration of Aβ of 1 μM. In Preparation B, Aβ from the stock solution was diluted by 12.5 mM NaOH in the volume ratio of 1:4, sonicated for 5 min and finally diluted by RB to obtain the final concentration of Aβ of 1 μM. In Preparation C, Aβ from the stock solution was mixed with HFIP in the volume ratio of 1:9, vortexed for 1 min and then the solvent was evaporated using a stream of nitrogen (solid Aβ was dissolved in RB to obtain the final concentration of Aβ of 1 μM).

### 4.2. Instrumentation

#### 4.2.1. Surface Plasmon Resonance (SPR) Biosensor

We used a six-channel SPR biosensor platform based on wavelength spectroscopy of surface plasmons (Plasmon VI) developed at the Institute of Photonics and Electronics, Prague. In this SPR platform, the angle of incidence of the light beam is fixed and changes in the resonance wavelength of surface plasmons are measured by analyzing the spectrum of polychromatic light reflected from an SPR chip. The resonance wavelength is sensitive to changes in the refractive index caused by the binding of biomolecules to the surface of an SPR chip. A shift in the resonance wavelength of 1 nm represents a change in the protein surface coverage of 17 ng/cm^2^. The SPR chips used in this study were prepared by coating microscope glass slides obtained from Marienfeld, Germany, with thin layers of titanium (1–2 nm) and gold (48 nm) via e-beam evaporation in vacuum. The SPR platform was combined with a dispersionless microfluidic module [42]. The active temperature stabilization unit allowed maintaining the temperature in the system with a precision of 0.01 °C. All experiments reported in this study were performed at a temperature of 25 °C and a flow rate of 20 µL/min.

#### 4.2.2. Functionalization of the SPR Chip

Prior to the experiments, the surface of an SPR chip was modified by a self-assembled monolayer of mixed thiols, on which specific antibodies (Ab(cypD) or Ab(17β-HSD10)) were immobilized using the amino-coupling method as described previously [43]. Briefly, a clean SPR chip was immersed in a 3:7 molar mixture of HS-C_11_-(EG)_6_-OCH_2_-COOH and HS-C_11_-(EG)_4_-OH (ethanol solution, total concentration of 0.2 mM), incubated for 10 min at 40 °C and then incubated for at least 12 h at room temperature in the dark. Prior to use, the chip was rinsed with ethanol and Milli-Q water, dried with a stream of nitrogen and mounted in the SPR platform. First, the mixture of 12.5 mM NHS and 62.5 mM EDC in Milli-Q water was injected (10 min) to activate the carboxylic groups. Then, Ab(cypD) or Ab(17β-HSD10) at a concentration of 10 μg/mL in SA10 was pumped through the flow cell until the response to the immobilized antibody leveled off (~15 min, surface coverage ~ 220 and 300 ng/cm^2^). Then, PBS_Na_ was applied (5 min) to remove the antibody non-covalently attached to the surface. Finally, 500 mM EA in Milli-Q water was injected (5 min) to deactivate the unreacted carboxylic groups. 

#### 4.2.3. Immobilization of cypD to the Functionalized SPR Chip

The SPR chip functionalized with Ab(cypD) was washed with MES. Then, the detection channels were exposed to 100 nM cypD in MES until the response to the immobilized cypD leveled off (~15 min, surface coverage ~100 ng/cm^2^), while the reference channels were kept in MES. Then, all channels were washed with MES (> 20 min) and exposed to PBS_Na_ (5 min) to remove the non-covalently attached cypD. 

In order to compensate for the variations in the surface coverage of the immobilized cypD after the PBS_Na_ washing, five different levels of cypD (surface coverage of 1.7–100 ng/cm^2^ before PBS_Na_ washing) were immobilized on the surface of an SPR chip. Then, MES was exchanged for RB containing 5 mM Mg^2+^ and 140 mM K^+^ and monomeric Aβ_1–40_ or oligomeric Aβ_1–42_ at a concentration of 1 μM in RB were injected in both the reference and detection channels (5 min). Finally, RB was injected again. The sensor responses obtained in the reference channels 5 min after switching back to the RB were subtracted from those obtained in the detection channels. Such reference-compensated sensor responses were plotted as a function of cypD surface coverage and fitted by the Boltzmann function. The experiment was repeated three times and the parameters obtained from the fitting were averaged and used to normalize the measured sensor responses to the binding of Aβ to varying levels of cypD to those corresponding to the binding of Aβ to cypD with the surface coverage of 34 ng/cm^2^.

#### 4.2.4. Immobilization of 17β-HSD10 to the Functionalized SPR Chip

The SPR chip functionalized with Ab(17β-HSD10) was washed with MES. Then, the detection channels were exposed to 100 nM 17β-HSD10 in MES until the response to the immobilized 17β-HSD10 leveled off (~15 min, surface coverage of 150 ng/cm^2^), while the reference channels were kept in MES. Then, all the channels were washed with MES (> 20 min). In contrast to the immobilization of cypD, the immobilization of 17β-HSD10 resulted in a stable surface that was resistant to changes in RB and thus no additional steps were necessary. 

### 4.3. Interactions of Aβ_1–40_ and Aβ_1–42_ with cypD and 17β-HSD10 at Different Concentrations of K^+^ and Mg^2+^

In all the experiments performed in this study, RB was injected into both the detection and reference channels of the functionalized SPR chip and flowed along the surface until the stable baseline was reached. Then, the sample of Aβ (prepared according to Preparations A–C, see Section 4.1.3 in Materials and Methods) at a concentration of 1 μM in the particular RB was injected into both the detection (surface with immobilized cypD or 17β-HSD10) and the reference (surface without immobilized cypD or 17β-HSD10) channels (5 min). Then, RB was injected again. The final sensor response was determined as a difference between the responses of the detection and reference channels 5 min after switching back to RB. The reference channel accounts for interferences (e.g., due to the electrostatically induced effects) and therefore the final reference-compensated sensor response corresponds to the specific binding of Aβ to cypD or 17β-HSD10 in the particular ionic environment. Each experiment was repeated at least three times and at least three sensor response values were used to calculate the mean and standard deviation of the sensor responses for the particular concentration of ions.

The effect of the ionic environment on the interactions of monomeric Aβ_1–40_ and oligomeric Aβ_1–42_ with cypD and 17β-HSD10 was investigated using RBs containing K^+^ at concentrations of 0, 15, 50, 140 and 500 mM and Mg^2+^ at concentrations of 0, 1, 5, 25 and 50 mM. The effect of the oligomerization state of Aβ on the interactions of Aβ with cypD was investigated using RBs containing 5 mM Mg^2+^ and 0, 15, 50, 140 and 500 mM K^+^ and Aβ_1–40_ and Aβ_1–42_ prepared in both monomeric and oligomeric forms.

## 5. Conclusions

In this work, we demonstrate that the ionic environment significantly affects the interactions between proteins of the mitochondrial matrix (cypD, 17β-HSD10) and Aβ and show that the particular ionic environment may increase the binding efficiency of these interactions in comparison with the physiological state. As the ionic environment in the mitochondrial matrix fluctuates in response to different physiological as well as AD-associated processes, its effect on the biomolecular interactions should be considered in the studies of the processes taking place in the mitochondrial matrix.
